# Exploring the Influences of BaO Amount on the Wettability and Mechanical Behavior of Vitrified Bond Diamond Composites

**DOI:** 10.3390/ma17020339

**Published:** 2024-01-10

**Authors:** Bingjian Guo, Haifeng Kuang, Xiaopan Liu, Hongyi Jiang, Rong Tu, Meijun Yang, Song Zhang

**Affiliations:** 1 School of Materials Science and Engineering, Wuhan University of Technology, 122 Luoshi Road, Wuhan 430070, China; bjguo@mtcn.net (B.G.); kuanghaifeng@whut.edu.cn (H.K.); jianghy@whut.edu.cn (H.J.); 2 Zhejiang MTCN Technology Co., Ltd., No. 59, Luhui Road, Taihu Street, Huzhou 311103, China; 3 Materials Science and Engineering, Hunan University, Changsha 410082, China; lxpchenmo@163.com; 4 State Key Laboratory of Advanced Technology for Materials Synthesis and Processing, Wuhan University of Technology, 122 Luoshi Road, Wuhan 430070, China; turong@whut.edu.cn (R.T.); kobe@whut.edu.cn (S.Z.); 5 Chaozhou Branch of Chemistry and Chemical Engineering Guangdong Laboratory, Chaozhou 521000, China

**Keywords:** BaO, vitrified bond, vitrified bond diamond composites, wettability, mechanical properties

## Abstract

In recent years, the vitrified bond diamond grinding wheel has been applied widely in automotive, aerospace and machine tools of manufacturing industries. However, the main problems of low intensity and poor wettability between the vitrified bond and diamond abrasive limit its further application. In this study, BaO was added into the basic SiO_2_–B_2_O_3_–Al_2_O_3_–R_2_O vitrified bond system, and the impact of BaO on the wettability, thermal and mechanical behavior of vitrified bond and vitrified bond diamond composites was systematically discussed, respectively. The test indicated that when the vitrified bond containing BaO of 6 wt.% was sintered with diamond abrasive at 750 °C, a continuous barium feldspar phase transition layer between diamond abrasive and the bond was generated, which ameliorated the wet property of the bond–diamond abrasive. The contact angle varied from 59° on the blank sample to 35°, and the expansion coefficient changed from 6.24 × 10^−6^/K to 5.30 × 10^−6^/K. The Rockwell hardness and flexural strength of the vitrified bond diamond composites achieved the peaks of 117.5 MPa and 113.6 MPa, respectively, which increased by 20.2% and 16.5% compared with that of sample without the addition of BaO.

## 1. Introduction

Diamond is often used as a grinding material for machining hard and brittle materials because of its high hardness [1,2], while vitrified bond is widely wielded as a bonding agent for diamond abrasive—because of its high elastic modulus, high chemical stability, strong self-sharpening and low grinding heat—to manufacture abrasive tools that must possess a precise shape and certain strength [3,4,5,6,7,8,9,10].

As the requirements of the manufacturing industry increase, the working speed of vitrified bond diamond abrasive tools is getting faster [11,12,13,14,15]. This means the vitrified bond used as a bonding material should not only have a higher intrinsic strength, but also a higher bond strength with diamond abrasive. According to the above demands, developments of vitrified bond with a lower refractoriness and an expansion coefficient matching the diamond abrasive, to ensure high strength of the vitrified bond diamond abrasive tools, has become a research hotspot in superhard material tool preparation [16,17,18].

Recently, scholars have performed much related research on the SiO_2_–B_2_O_3_–Al_2_O_3_–R_2_O vitrified bond, which is extensively wielded in the preparation of diamond abrasive— its simple smelting process, low raw material cost and high intrinsic mechanical strength make it capable of achieving the low-temperature sintering of the system’s bond [19,20,21,22,23,24,25]. Some scholars have found the addition of ZrO_2_ can improve the flexural strength and reduce its thermal expansion coefficient, while a small amount of ZrO_2_ greatly increases the refractoriness [20,21,22]. It has been reported that V_2_O_5_ can enhance the wet property of the bond–diamond abrasive [23], but it reduces the strength and increases the expansion coefficient of the bond [24].

BaO has been regarded as an effective addition to glass–ceramics systems, which can significantly change the refractoriness and thermal expansion coefficient via influencing the kind and character of the crystalline phases present [26,27]. Thus, BaO can provide the possibility to enhance the wet property of the bond–diamond abrasive, as well as the performance. FNML was used to investigate the effect of the addition of BaO on the crystallization temperature and phase transition of Li_2_O–Al_2_O_3_–ZnO–SiO_2_–TiO_2_–ZrO_2_ (LAZSTZ) glass [28]. However, the impacts of BaO with various contents on the wettability, thermal and mechanical behavior of vitrified bond and vitrified bond diamond composites have hardly been systematically studied.

In this work, both BaO-containing and BaO-free SiO_2_–B_2_O_3_–Al_2_O_3_–Li_2_O vitrified bond systems are composed, as well as the vitrified bond diamond composites. Thermal characteristics and crystallization performance of the vitrified bond are methodically displayed. The bonding state generated between the vitrified bond and the diamond are studied, and the flexural strength of the vitrified bond diamond composites is tested and depicted.

## 2. Materials and Methods

### 2.1. Sample Preparation

The basic binder was composed of SiO_2_, B_2_O_3_, Al_2_O_3_ and Li_2_O at a mass ratio of 64:16:12:8. And 0, 2, 4, 6, 8, 10 wt.% BaO from the corresponding contents of BaCO_3_ were added into the basic binder and mixed evenly to form BaO–SiO_2_–B_2_O_3_–Al_2_O_3_–Li_2_O mixed powder, respectively. The mixed powder was put into the crucible furnace and then was heated to 1200 °C at a speed of 3 °C/min. After holding for 2 h, the molten bond was quenched with cold water to obtain bond glass fragments. The fragments were milled in milling machine for 12 h. Then, the milled vitrified bond powders were pressed to the molding under the pressure of 70 MPa for 2 min to fabricate a cuboid test strip (40 mm × 6 mm × 5 mm); the cuboid test strip was heated to 750 °C, surrounded with nitrogen for 2 h and then cooled down to room temperature to obtain vitrified bond test strips containing different BaO contents.

The milled vitrified bond powder, diamond (Henan Yellow River Cyclone Co., Ltd., Changge, China, HMB diamond, particle size 85~110 μm) and temporary vitrified bond PVA were mingled homogeneously at a mass ratio of 20:75:5 and subsequently compressed to form test strips (40 mm × 5 mm × 6 mm) and cylindrical samples (φ20 mm × 6 mm) at 70 MPa for 2 min. After drying for 24 h at room temperature, the preformed samples were heated to 750 °C for 2 h in argon atmosphere, and after cooling to room temperature, the strip and cylindrical vitrified bond diamond composite samples were obtained.

### 2.2. Sample Characterization

X-ray polycrystalline powder diffractometer (XRD, Philips, Amsterdam, Netherlands, PW1420, CuKα radiation, 30 kV, 40 mA) was applied to test the phase of the vitrified bond. Scanning electron microscopy (SEM, TESCAN, MIRA3 LMH, Brno, Czech) with EDS was utilized to obtain the composition and morphology of the vitrified bond samples, as well as the composites. Dilatometry (DIL402SE) was used to gauge thermal expansion coefficient of vitrified bond from room temperature to 750 °C. And the maximum deformation temperature during this processing was regarded as the refractoriness of the vitrified bond. The Plane flow (Seger Tone) method was applied to characterize the high-temperature fluidity of the bond cylinder by placing the bond cylinder with a diameter of 8 mm on a ZrO_2_ substrate in a muffle furnace (SX2–8–17T). Then, the final diameters of the bond cylinder were measured after being heated at different temperatures (650 °C, 670 °C, 690 °C, 710 °C, 730 °C, 750 °C, 770 °C) for 1 h. At last, the ratio of the final diameter to the initial diameter (8 mm) reflected the fluidity [29]. The electronic universal material testing machine (INSTRON-3382, Boston, MA, USA) was arranged to obtain the flexural strength of the sintered vitrified bond together with the diamond composite samples through three-point bending method. The hardness of vitrified bond diamond composite test strips was tested through HR-150DT Rockwell hardness tester (Shanghai Materials Testing Factory, Shanghai, China) with quenched steel ball of φ1.59 mm and a load of 980 N.

Since diamond abrasives are granular, the high temperature wettability of vitrified bond and diamond cannot be measured quantitatively. Referring to the relevant literature [30], the vitrified bond cylinders with different formulations were placed on a monocrystalline silicon sheet coated with diamond film, and the high-temperature wettability angle of bond–diamond at 750 °C was gauged by high-temperature contact angle tester (JC2000D2, Beijing Zhongyi kexin Technology Co., Ltd., Beijing, China).

## 3. Results and Discussion

### 3.1. Phases and Microstructure of the Vitrified Bond

**Figure 1:**
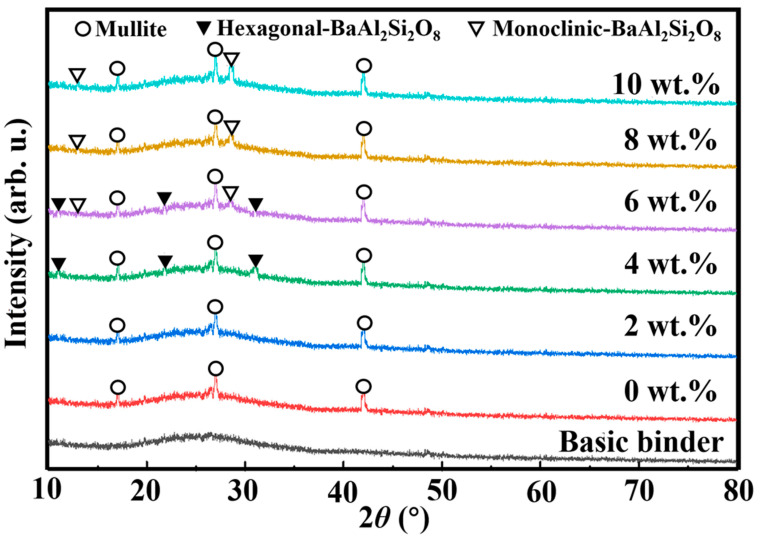
XRD of vitrified bonds with different BaO contents after sintering at 750 °C for 2 h.

Figure 1 displays the XRD patterns of vitrified bonds containing various BaO contents. There is a pure glass phase without any diffraction peak due to the fast cooling speed of the basic binder; thus, a smooth wave packet appears within the range of 20–35°. After the basic binder is heat-treated at 750 °C at 0 wt.% (Figure 1), the mullite phase (3Al_2_O_3_ · SiO_2_) appears in the sample. When a small amount of BaO (2 wt.%) is added to the vitrified bond and there is no obvious influence on the crystallization behavior of the vitrified bond, the mullite phase is precipitated as before, and the intensity of the diffraction peak does not change. With the content of BaO in the vitrified bond reaching to 4 wt.%, the mullite phase still remains in the vitrified bond, while the hexagonal barium feldspar phase (BaO · Al_2_O_3_ · 2SiO_2_) begins to precipitate, too. When one continues to increase the content of BaO to 6 wt.%, the mullite phase, hexagonal barium feldspar phase and monoclinic barium feldspar phase appear together inside the vitrified bond, and the diffraction maximum intensity of the hexagonal barium feldspar phase decreases. When the content of BaO reaches to 8 wt.%, only the mullite and monoclinic barium feldspar phases appear in the XRD spectrum, and the hexagonal barium feldspar phase completely disappears. If one continues to increase the content of BaO to 10 wt.%, only the mullite phase and monoclinic barium feldspar phase are still obviously in the XRD spectrum, and the diffraction peak intensity of the monoclinic barium feldspar phase is significantly enhanced.

**Figure 2:**
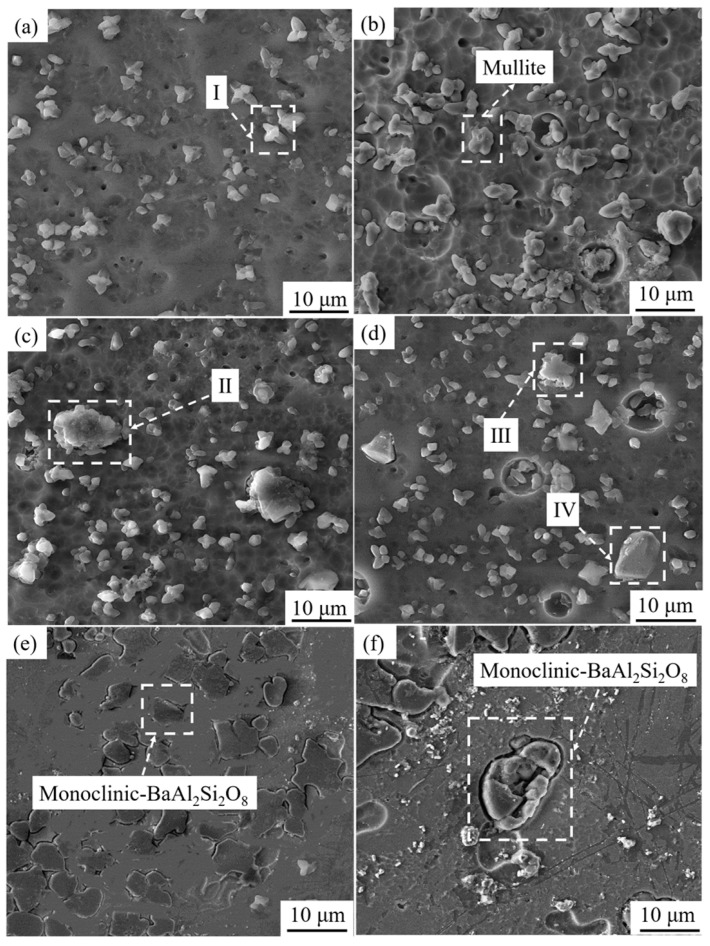
Microstructure of vitrified bonds with different BaO contents: (**a**) 0 wt.%; (**b**) 2 wt.%; (**c**) 4 wt.%; (**d**) 6 wt.%; (**e**) 8 wt.%; (**f**) 10 wt.%.

Figure 2 and Table 1 depict the cross-section morphology and EDS analysis of precipitated crystals after the corrosion of vitrified bonds with different BaO contents. In Figure 2a, mullite crystal of 1–5 μm appears and distributes in the sample without BaO.

When the content of BaO is 2 wt.%, there is no obvious influence on the crystallization behavior of the vitrified bond and the mullite phase is precipitated as before (Figure 2b), which is consistent with the result of the XRD pattern (Figure 1).

When the content of BaO is 4 wt.%, hexagonal barium feldspar grains with a grain size about 10 μm begin to precipitate, and the morphology presents a layered structure of multiparticle accumulation, as indicated in area II (Figure 2c).

When the content of BaO is 6 wt.%, monoclinic barium feldspar grains begin to appear in the vitrified bond, as shown in area IV (Figure 2d), and its crystal morphology shows equal integration, which is obviously different from the hexagonal barium feldspar crystals [31,32]; the crystal size is 5–10 μm.

When the content of BaO is 8 wt.% (Figure 2e), the fracture surface of the bond is all monoclinic barium feldspar grains, and the number of crystals increases significantly.

When the content of BaO reaches 10 wt.% (Figure 2f), the monoclinic barium feldspar grains develop secondary crystallization, and the grain size increases significantly.

### 3.2. Crystallization Mechanism of Vitrified Bonds with Different Content of BaO

From the XRD pattern (Figure 1) and microstructure (Figure 2) of the vitrified bonds, the crystallization mechanism of vitrified bonds containing different BaO can be illustrated as follows: In the basic binder, the LiO_2_ in the vitrified bond will provide free oxygen to [AlO_6_]^6^ and lead itself to be transformed into [AlO_4_]^4^, which will connect with [SiO_4_]^4^ [33]. And Li^+^ fills in the network gap around Al^3+^ to ensure local electrical neutrality. At the same time, as the radius of Li^+^ is small and the outermost electron is 2, Li^+^ has a strong polarization effect on the surrounding oxygen ions, generating a large number of [SiO_4_]^4−^ structures with low degrees of association, so as to promote the phase separation of the vitrified bond and precipitate the mullite phase [34].

When a small amount of BaO (2 wt.%) is added, BaO will provide “free oxygen” to the structure of the vitrified bond network, which will interrupt the bridge oxygen in the silicon oxygen tetrahedron and destroy the integrity of the vitrified bond network, thereby reducing the association degree of [SiO_4_]^4−^ and the viscosity of the vitrified bond. This promotes the diffusion of cations [35] and improves the phase separation tendency of the vitrified bond but will not cause the new crystal phase precipitation.

When the content of BaO reaches 4 wt.%, the water-quenched bond after smelting is a pure glass phase, which contains a large number of broken chain silicon oxide tetrahedrons and aluminum oxide tetrahedrons. The gap of the bond network also contains more Ba^2+^ that tends to attract non-bridge oxygen ions to the surrounding structure, which will improve the free energy of the whole bond network structure, so that the glass phase matrix of the bond begins to produce phase separation. In addition, during the sintering, Ba^2+^ will cause the surrounding silicon oxygen tetrahedron and aluminum oxygen tetrahedron to rearrange, forming a hexagonal layered structure filled with Ba^2+^ between layers, forming hexagonal barium feldspar crystals [36].

Thermodynamically, when the temperature is below 1590 °C, monoclinic barium feldspar is a thermodynamic stable phase. However, because the order of the Si^4+^ and Al^3+^ arrangement in the two-dimensional layered structure of hexagonal barium feldspar is higher than that of the Si^4+^ and Al^3+^ arrangement in the three-dimensional network of monoclinic barium feldspar in the vitrified bond, the hexagonal barium feldspar phase is preferentially precipitated [37].

With the amount of BaO content raised (6 wt.%), the integrality of the three-dimensional network structure in the glass phase of the vitrified bond worsens, and the attraction of Ba^2+^ with more content to non-bridge oxygen is strengthened. At the same time, the diffusion of Si^4+^ and Al^3+^ increases during the sintering process, further driving the glass phase vitrified bond to precipitate crystals, making the SiO_2_ and Al_2_O_3_ tetrahedral structure around Ba^2+^ form a three-dimensional spatial network, while Ba^2+^ is in the network gap of eight tetrahedrons [38], so the thermodynamic stable monoclinic barium feldspar phase starts to precipitate in the vitrified bond, which will enhance the wet property and depress the thermal expansion of the vitrified bond diamond composites.

### 3.3. Impact of BaO on Thermal Behavior of the Vitrified Bond

**Figure 3:**
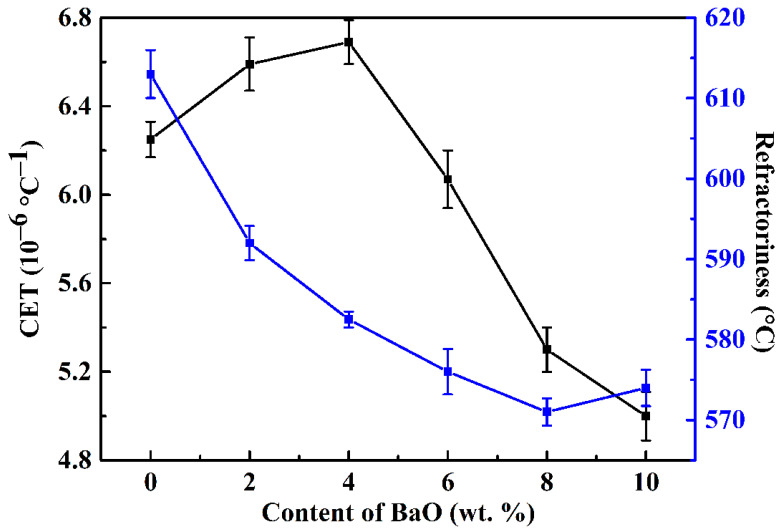
Effect of BaO on expansion coefficient and refractoriness of vitrified bond.

Figure 3 shows the impact of BaO on the refractoriness and expansion coefficient of the vitrified bond. When BaO is not added, the expansion coefficient of the foundation bond is 6.24 × 10^−6^/K. When the content of BaO is 2–4 wt.%, the expansion coefficient of the binder increases slightly due to the added precipitates of BaO in the form of hexagonal barium feldspar crystals (Figure 2a), whose expansion coefficients are 8.02 × 10^−6^/K, which is greater than the base binder [31]. When the content of BaO is 4–10 wt.%, hexagonal barium feldspar crystals and monoclinic barium feldspar crystals begin to precipitate simultaneously; the expansion coefficient of monoclinic barium feldspar is 2.3 × 10^−6^/K [39], which is far less than the basic binder, so the expansion coefficient of the binder falls greatly with more precipitation of monoclinic barium feldspar crystals; when the BaO content is 10 wt.%, it decreases to 4.98 × 10^−6^/K.

When BaO is not added, the refractoriness of the base binder is 613 °C; when the content of BaO is 0–8 wt.%, the refractoriness of the binder decreases due to the “free oxygen” provided by BaO, which breaks the chains, so reducing the integrality of the three-dimensional network structure of the binder [40]. When the content of BaO is increased to 10 wt.%, the refractoriness rises slightly to 574 °C due to more monoclinic barium feldspar crystals being precipitated from the binder.

**Figure 4:**
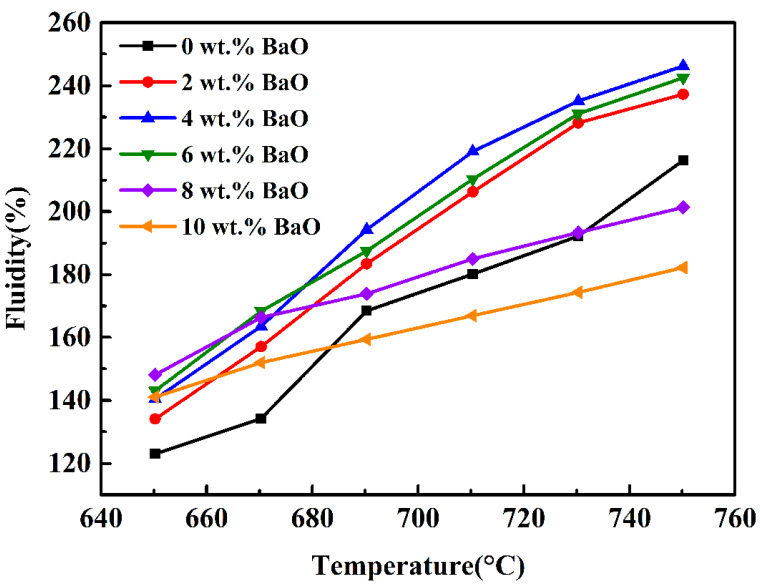
Effect of BaO on fluidity of vitrified bond at different sintering temperatures.

Figure 4 shows the relationship between the fluidity and the contents of BaO of the vitrified bond at different sintering temperatures. It indicates that the fluidity of each group of vitrified bonds increases when the sintering temperature is increased. When the content of BaO is ≤4 wt.%, the proportion of non-bridging oxygen provided by BaO in the glass phase matrix of the vitrified bond increases with more BaO added, which destroys the integrality of its three-dimensional network structure [40], thus promoting the flowability of the vitrified bond. Outside this range, not only does the structure of the glass phase inside the vitrified bond become more fragile with the increase of BaO content, but the barium feldspar phase is precipitated in the vitrified bond due to part of Ba^2+^ when sintering at the flow-test temperature. When sintered at 650 °C, as the higher viscosity of the bond is not conducive to the rearrangement of silicon oxide tetrahedron and aluminum oxide tetrahedron around Ba^2+^, there is less barium feldspar precipitated in the bond, and the fluidity of the bond has a peak value of 148% when the content of BaO is 8 wt.%. When sintered at more than 650 °C, the decrease of the vitrified bond viscosity promotes more barium feldspar crystals to precipitate, thus hindering the flowability of the vitrified bond.

### 3.4. Impact of BaO on the Wet Property of Vitrified Bond Diamond Composites

**Figure 5:**
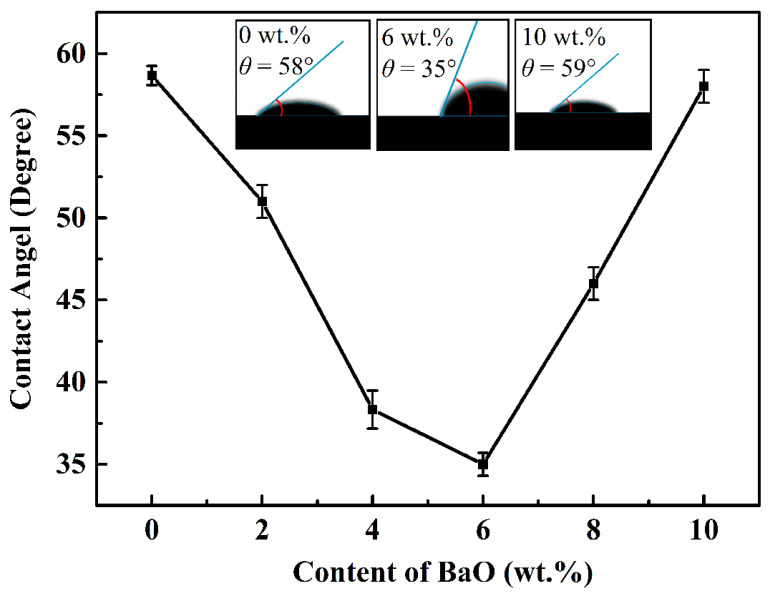
Effect of BaO on contact angle between bond and diamond film.

The impact of the BaO contents on the contact angle of the bond–diamond abrasive after sintering at 750 °C is revealed in Figure 5. The vitrified bond with 0 wt.% BaO can wet the diamond film with a contact angle of 59°. When the content of BaO increases, the contact angle decreases rapidly, reaching a minimum of 35° when the content of BaO is 6 wt.%. However, should the content of BaO continue to increase, the contact angle increases again to 58° when the content of BaO is 10 wt.%.

**Figure 6:**
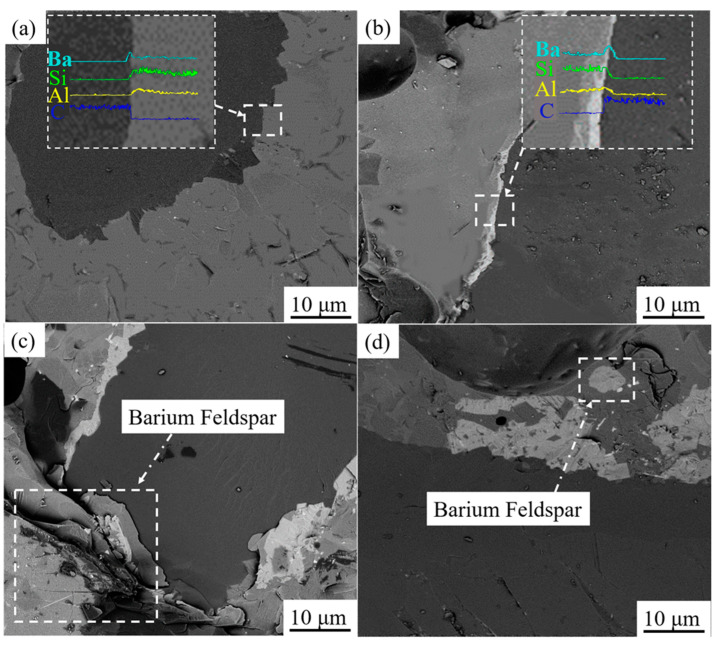
Interface morphology of vitrified bond and diamond abrasive with different BaO content: (**a**) 2 wt.%; (**b**) 4 wt.%; (**c**) 6 wt.%; (**d**) 8 wt.%.

Figure 6 shows the interface between bonds with different BaO content and the diamond abrasive after sintering at 750 °C. After sintering, the bond containing 2 wt.% BaO has good wettability with the diamond abrasive; the interface is clear and compact, with no new phase observed at the interface, and the EDS analysis at the interface shows that there is an enrichment of the Ba element between the bond and diamond. When the content of BaO reaches 4 wt.%, a thin white transition layer with a thickness of less than 3 μm appears at the interface of the bond and diamond. The EDS analysis at the interface shows that the transition layer is rich in Ba and Al elements. Combined with the XRD spectrum of the sintered vitrified bond in Figure 1, it is determined that the transition layer is the barium feldspar phase precipitated at the interface. When the content of BaO increases to 6 wt.%, the barium feldspar phase transition layer at the interface is obviously thickened, and the local thickness reaches 10 μm; when the content of BaO reaches 10 wt.%, the continuous barium feldspar transition layer is no longer formed at the interface, but larger barium feldspar crystals are precipitated at the local interface and inside the vitrified bond.

**Figure 7:**
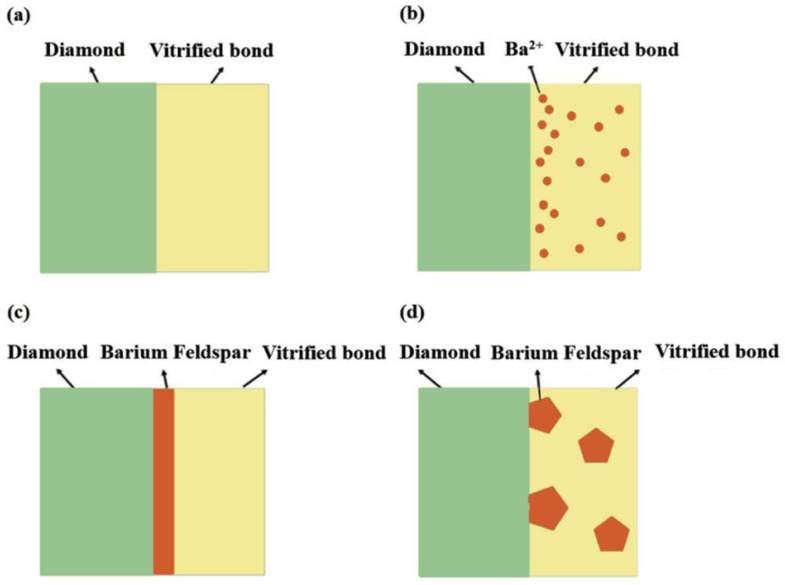
Schematic diagram of interface between vitrified bond and diamond abrasive with different BaO content: (**a**) 0 wt.%; (**b**) <4 wt.%; (**c**) 4–6 wt.%; (**d**) >6 wt.%.

Combined of Figure 5 and Figure 6 results, the influence mechanism of BaO on the wet property of bond–diamond abrasive can be exhibited in Figure 7. When the content of BaO appears low, the vitrified bond melts into a viscous fluid at the 750 °C sintering temperature and Ba^2+^ also obtains a high diffusion rate. Furthermore, the surface of the diamond abrasive presents a negative potential [41], and Ba^2+^ will migrate to the diamond interface under the effect of electrostatic attraction, thus enriching at the diamond surface. Ba^2+^at the diamond surface will also attract O_2_ from the vitrified bond, finally improving the wet property of the bond–diamond abrasive. When the content of BaO reaches 4 wt.%, the content of Ba^2+^ on the diamond abrasive surface is higher. Consequently, the concentration required for barium feldspar crystallization will be reached first, and fine barium feldspar grains will be precipitated on the diamond surface, forming a continuous barium feldspar precipitation layer. The diamond abrasive is an atomic crystal constructed by combining C atoms with *sp*^3^ hybrids, and its surface chemical activity is low [42], while the formation of the barium feldspar precipitation layer on the diamond abrasive surface can considerably increase the wet property of the bond–diamond abrasive. With more BaO dropped, the thickness of the barium feldspar precipitation layer on the diamond abrasive surface increases, and the contact angle of the bond and the diamond abrasive constantly fall. When the content of BaO reaches 8 wt.%, the concentration of Ba^2+^ contained in the glass phase vitrified bond before sintering can reach the concentration required for barium feldspar crystallization without enrichment on the diamond surface. Therefore, barium feldspar starts to crystallize on the local surface of the diamond and inside the vitrified bond at the same time during sintering. Large barium feldspar crystals only precipitate locally from the diamond, which means no continuous barium feldspar precipitation layer is formed on its surface. On the other hand, due to the large amount of monoclinic barium feldspar crystals precipitated in the bond, the fluidity of the bond during sintering at 750 °C is greatly reduced (Figure 4), leading to a fall in the wet property of the bond–diamond abrasive.

### 3.5. Effect of BaO on Mechanical Behavior of Vitrified Bond Diamond Composites

**Figure 8:**
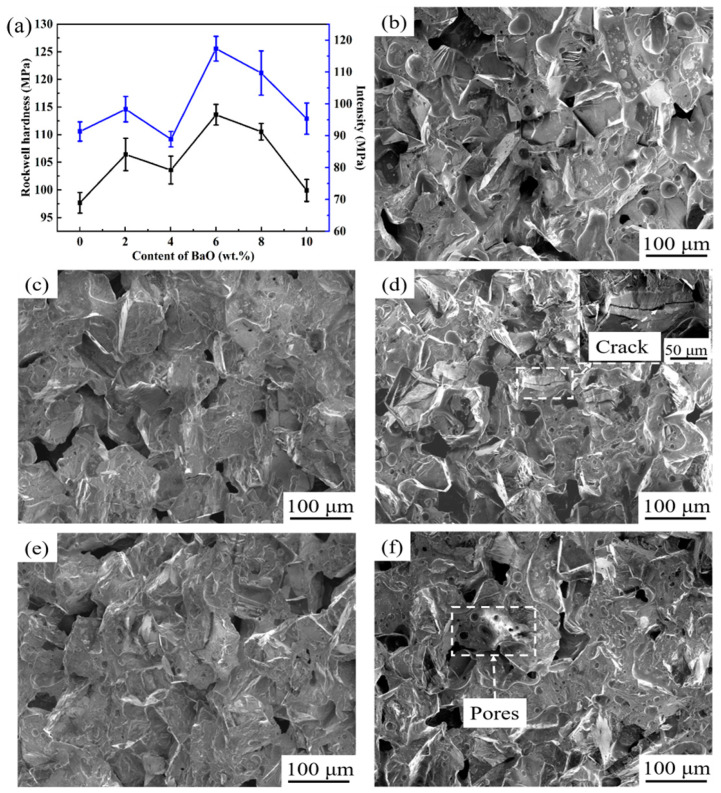
Effect of BaO on mechanical properties (**a**) and fracture morphology of vitrified bond diamond composites: (**b**) 0 wt.%; (**c**) 2 wt.%; (**d**) 4 wt.%; (**e**) 6 wt.%; (**f**) 10 wt.%.

Figure 8 shows the impact of BaO on the mechanical behavior and fracture morphology of vitrified bond diamond composites, respectively. After being sintered at 750 °C, the vitrified bond of the 0 wt.% content sample presents a completely molten state, which fills in the pores between the diamond abrasives and encloses most of them, but some of the diamond abrasives are still uncoated (Figure 8a). When the content of BaO reaches to 2 wt.%, although the addition of BaO increases the expansion coefficient of the vitrified bond and reduces its intrinsic strength, which is unfavorable for enhancing the flexural strength of the composite, the addition of BaO improves the wet property of the bond and the diamond as well as its fluidity during sintering, making itself owing a good encapsulation of the diamond after sintering. Thus, the diamond abrasive surface at the fracture is coated with molten vitrified bond (Figure 8b), which improves the hardness and intensity of the vitrified bond diamond composite.

When the amount of BaO reaches 4 wt.%, although the fluidity of the bond and the wet property with the diamond abrasive are further improved, the expansion coefficient of the bond reaches its maximum value of 6.24 × 10^−6^/K, resulting in poor thermal compatibility with the diamond abrasive. There is a large thermal stress in the sintered composite material, causing local stress cracks in the sample (Figure 8c), so the hardness and intensity of the sample begin to decline. When the content of BaO is increased to 6 wt.%, the vitrified bond maintains good fluidity, and the contact angle with diamond is also the minimum. At the same time, the crystalline phase precipitated in the vitrified bond begins to transform from the hexagonal barium feldspar phase with a high expansion coefficient to the monoclinic barium feldspar phase with a low expansion coefficient, and the expansion coefficient of the vitrified bond falls rapidly, which enhances the thermal compatibility of the vitrified bond and diamond abrasive. As such, the intensity and hardness increase rapidly to 117.5 MPa and HRB113.6, respectively; they increase by 20.2% and 16.5% compared with the 0 wt.% content sample.

If the content of BaO in the bond continues to increase, the content of the monoclinic barium feldspar phase precipitated in the bond after sintering increases, and the expansion coefficient decreases. However, exceeding the monoclinic barium feldspar phase reduces the fluidity of the bond at the sintering temperature (Figure 5), further affecting the densification of the bond. For example, when the content of BaO in the sintered bond is 10 wt.%, there are a lot of pores (Figure 8e), which reduces the hardness and intensity of the sample.

## 4. Conclusions

1. The change of the amount of BaO can control the phase transformation of barium feldspar in the vitrified bond, thus adjusting its refractoriness and thermal expansion coefficient. When the content of BaO is higher than 4 wt.%, according to the precipitation of the monoclinic barium feldspar phase, the expansion coefficient of the bond drops to 4.98 × 10^−6^/K; when the content of BaO reaches 10 wt.%, it matches well with the diamond abrasives.
2. When the vitrified bonds encompassing 4–6 wt.% BaO are sintered with the diamond abrasive at 750 °C, a continuous barium feldspar phase transition layer emerges at the bond–diamond abrasive interface, increasing the wet property of the bond–diamond abrasive.
3. When more BaO is added, the Rockwell hardness and flexural strength of the vitrified bond diamond composites rise firstly and fall subsequently. When the addition of BaO is 6 wt.%, maximums of 117.5 MPa and HRB113.6 are obtained, respectively, which increase by 20.2% and 16.5% compared with that of the sample without added BaO. This illustrates that BaO in vitrified bonds can certainly improve the mechanical behavior of diamond abrasives.

## Figures and Tables

**Table 1:** EDS analysis of vitrified bonds with different BaO contents.

Element	I		III		IV	
	wt.%	at.%	wt.%	at.%	wt.%	at.%
O	47.32	60.46	33.05	60.31	34.07	61.44
Al	38.75	29.34	14.46	15.64	14.37	15.36
Si	13.93	10.17	15.51	16.17	15.05	15.51
Ba	-	-	36.98	7.88	36.51	7.69

## Data Availability

Data are contained within the article.
